# Editorial: Artificial intelligence and mental health care

**DOI:** 10.3389/fpubh.2024.1461446

**Published:** 2024-07-30

**Authors:** Jorge P. Simões, Peter ten Klooster, Patrick K. Neff, Uli Niemann, Jannis Kraiss

**Affiliations:** ^1^Department of Health Psychology and Technology, University of Twente, Enschede, Netherlands; ^2^Department of Otorhinolaryngology, Head, and Neck Surgery, University Hospital Zurich, University of Zurich, Zurich, Switzerland; ^3^Department of Psychiatry and Psychotherapy, University of Regensburg, Regensburg, Germany; ^4^University Library, Otto-von-Guericke-University Magdeburg, Magdeburg, Germany

**Keywords:** mental health, healthcare, public health, machine learning, artificial intelligence

## Introduction

Advancements in machine learning (ML) and artificial intelligence (AI) offer significant potential to transform mental health care. These technologies have been utilized for various purposes, such as early detection of mental disorders, optimizing personalized treatments tailored to individual patient characteristics, improving the characterization of disorders that negatively impact mental wellbeing and quality of life, better predicting their progression over time, and developing new treatments and diagnostic tools for mental health care. Despite their considerable potential and occasional breakthroughs, ML and AI have not yet fully realized these objectives in mental health care.

## Aim of this Research Topic

This Research Topic aimed to provide innovative examples of how ML and AI applications can be practically implemented in standard mental health care. The particular focus of this Research Topic was to provide examples of how to use ML and AI to enhance public health by lessening the impact of chronic disorders that adversely affect wellbeing and improving quality of life.

## Research Topic impact

This Research Topic was open between November 10th, 2022, and November 1st, 2023. There were 14 submissions, 12 of which were accepted after peer review, from 64 different authors. While open, the topic had 26,973 views, 19,768 article views, 5,845 article downloads, and 1,360 topic views.

Alsaqqa and Alwawi conducted a scoping review on the characteristics of studies, related concepts, and recommendations for implementing digital interventions in public health. It highlighted the importance of addressing structural inequalities, ensuring personal agency, and social connectedness. The study also emphasized the importance of iterative optimization during study design, involving stakeholders, and using contextual indicators to enhance the effectiveness of digital interventions. An important aspect of the review was the call for more patient and public involvement and the suggestion to adopt standardized metrics to improve research quality and application of digital health interventions.

Morita et al. explored the application of large language models like ChatGPT in public health through SWOT and PESTLE analyses. The identified strengths include personalized health support and data analysis capabilities, weaknesses such as potential miscommunication and data privacy issues, opportunities in improving healthcare access and disease surveillance, and threats including misinformation and bias. The PESTLE analysis identified factors like government policies impacting investment and data governance, cost-effectiveness and job impact considerations, public trust and cultural attitudes toward AI, integration with health systems and algorithmic transparency, privacy laws and ethical guidelines, and the environmental impact of AI infrastructure's energy consumption and carbon footprint.

Wen et al. used 2D gait videos for automatic anxiety assessment among graduate students. By analyzing gait features from time-series data, the authors created anxiety assessment models via machine learning. The study found that dynamic time-frequency features significantly enhance model performance, particularly for women. The models demonstrated reliability and validity, suggesting that 2D gait analysis could be a practical, non-invasive method for real-time anxiety assessment and should be further investigated and evaluated in clinical samples.

Huisman et al. examined the validity of automated sentiment analysis in interpreting emotional content from therapy session notes of patients with eating disorders, comparing it to human raters. The study analyzed 460 records and found moderate agreement between automated analysis and human raters. The findings suggest the potential for automated sentiment analysis in clinical settings but emphasize the need for further refinement before applying the algorithm in clinical settings, particularly by incorporating ED-specific terminology and establishing more relevant benchmarks for validation.

Franken et al. investigated the ability of ML to predict improvement in patients using real-world longitudinal data from specialized outpatient mental health treatment. Different ML models were trained and compared with traditional logistic regression. The models showed moderate predictive ability in an independent test set, with slightly better performance when early change scores were included as predictors. Machine learning algorithms did not outperform simpler logistic regression models. Early change during treatment was a crucial predictor for longer-term outcomes.

Li et al. also aimed to leverage the advantages of an ML approach over traditional statistical methods to predict the risk of depression in people with obstructive sleep apnea hypopnea syndrome using data readily available from the NHANES database. Several features predictive of depression were identified, including demographic, health and lifestyle-related, and socio-economic factors. Interestingly, like in the study by Franken et al., the simple logistic regression model was not inferior—and even superior—to more complex ML models.

Kim et al. used ML methods to examine the performance of classifying states of stress and non-stress using biosignal data measured by a smartwatch. In contrast to the previous studies, this study used an experimental setup where participants were instructed to perform stress-inducing and relaxation tasks. The top 9 features extracted from the heart rate and photoplethysmography data were able to classify stress with an accuracy of >80% with, again, the logistic regression classifier showing the best performance.

Delgadillo et al. performed a study during the COVID-19 pandemic using Bayesian network analyses and modeling interactions between risk and protective factors for suicidal ideation in Austria and the UK. The models achieved high predictive accuracy (AUC ≥ 0.84 within-sample and AUC ≥ 0.79 out-of-sample), explaining nearly 50% of suicidal ideation variability. Seven consistent factors, including depressive symptoms, loneliness, and anxiety, were identified in both countries. This study shows the potential to predict suicidal risk accurately using these factors.

Jović et al. addressed the challenge of comparing ADHD scores across different scales used by various research consortia. They harmonized scores from the Child Behavior Checklist (CBCL) and Strengths and Difficulties Questionnaire (SDQ) using various test equating and machine learning methods on 1,551 parent reports of children aged 10–11.5 years. The study found that methods utilizing item-level information and treating outcomes as interval measurements, such as regression, were most effective for harmonizing scores.

Pavicic et al. used iterative Random Forests to identify geographic, environmental, and sociodemographic predictors of suicide attempts among U.S. veterans. Analyzing data from 405,540 patients, the model incorporated 1,784 features, including climatic factors, population demographics, and the density of firearms and alcohol vendors. Key findings indicated that areas with higher concentrations of married males have lower suicide attempt rates, while areas with renting and males living alone have higher rates.

Bremer-Hoeve et al. investigated predictors of treatment dropout in patients with post-traumatic stress disorder (PTSD) due to childhood abuse, using elastic net regression. Analyzing data from 121 patients undergoing two different Eye Movement Desensitization and Reprocessing (EMDR) therapy protocols, they identified key dropout predictors: male gender, low education, suicidal thoughts, emotion regulation issues, high general psychopathology, and lack of benzodiazepine use.

Guo et al. explored causal factors of non-suicidal self-injury (NSSI) in children using computational causal analysis. They identified nine key factors: life satisfaction, depression, family dysfunction, sugary beverage consumption, positive youth development (PYD), internet addiction, COVID-19 PTSD, academic anxiety, and sleep duration. The research highlighted four main causal pathways and emphasized the roles of pandemic-induced lifestyle changes, screen time, adolescent development, and family dynamics in NSSI risk, advocating for targeted interventions addressing these diverse factors.

## Author contributions

JS: Writing – review & editing, Writing – original draft. PK: Writing – review & editing, Writing – original draft. PN: Writing – review & editing, Writing – original draft. UN: Writing – review & editing, Writing – original draft. JK: Writing – review & editing, Writing – original draft.

